# Materiovigilance: Knowledge, attitude, practice among healthcare professionals in India

**DOI:** 10.6026/973206300223581

**Published:** 2026-06-30

**Authors:** Hemlata Verma, Prerna Jasmine Kerketta, Shilpa Jain, Mahendra Soni

**Affiliations:** 1 Department of Pharmacology, Gandhi Medical College, Bhopal, Madhya Pradesh, India; 2 Department of Pharmacology, People's College of Medical Sciences & Research Centre, Madhya Pradesh, Bhopal, India; 3 Department of Hospital Administration, Gandhi Medical College, Bhopal, Madhya Pradesh, India

**Keywords:** Medical device, health care professionals, patient safety, tertiary care center, cross-sectional study

## Abstract

Despite 636 Medical Device Monitoring Centers (MDMCs) in India, post-marketing vigilance for medical devices remains less developed than for drugs. An institutional survey among health care professionals (HCPs) including consultants, physicians, nurses and midwives was conducted using a pre-validated electronic questionnaire. Among 107 respondents (36% response rate), 58% had heard of 'materiovigilance', 47.6% knew the MDAE reporting pathway and 14.9% had reported adverse events. Majority 96.2% agreed that MDAE reporting enhances patient safety and 89.2% supported its inclusion in undergraduate medical curricula. HCPs exhibited a positive attitude towards materiovigilance but knowledge gaps and suboptimal reporting persist; training and stronger reporting systems can promote materiovigilance activities and improve patient safety.

## Background:

India's medical device industry, currently valued at nearly USD 14 billion, is experiencing rapid growth and is projected to reach approximately USD 30 billion by 2030. As one of the top 20 medical device markets globally, the sector is expanding due to rising healthcare demands, technological advancements and supportive governmental initiatives [1]. The increasing utilization of medical devices in diagnosis, treatment and patient care has also resulted in a corresponding rise in medical device- associated adverse events (MDAEs), emphasizing the importance of effective surveillance systems [2, 3]. To strengthen the monitoring of MDAEs, the Government of India launched the Materiovigilance Programme of India (MvPI) in 2015 under the supervision of the Drugs Controller General of India (DCGI), with coordination by the Indian Pharmacopoeia Commission (IPC) [4]. The World Health Organization (WHO) defines materiovigilance as the systematic process of identifying, collecting, reporting and analysing adverse events related to medical devices to improve patient safety and prevent recurrence [5]. In addition to monitoring device-related adverse events, MvPI also aims to enhance awareness and reporting practices among healthcare professionals [6]. To facilitate reporting across the country, Medical Device Adverse Event Monitoring Centres (MDMCs) have been established under the programme, creating a nationwide network for medical device safety surveillance [7]. However, despite these initiatives, the post-marketing surveillance system for medical devices in India remains less developed than pharmacovigilance systems. Limited awareness, inadequate training and underreporting among healthcare professionals continue to hinder the effective implementation of materiovigilance. Furthermore, the wide diversity in the design, application and risk profiles of medical devices poses additional challenges in adverse event monitoring [8]. Therefore, it is of interest to evaluate the knowledge, attitude and practices (KAP) regarding materiovigilance among healthcare professionals in a tertiary care centre in Central India and to identify areas for improving its integration into routine clinical practice.

## Materials and Methods:

A cross-sectional, questionnaire-based study was conducted among healthcare professionals (50 specialists, 30 midwifery staff, 20 medical officers and 200 nursing staff) in a tertiary care hospital of Central India. Electronic forms with pre-validated questionnaires were used [7, 8]. Participants could fill the form after giving informed consent. A total of 15 questions related to the knowledge, attitude and practice of materiovigilance were asked. Data was analysed using descriptive statistics and results were computed. Ethical clearance was obtained from the Institutional Ethics Committee, before starting the study (Protocol No.: 208/IEC/2024 dated 13.12.2024)

## Study duration:

Three months (data collection in Month 1; analysis and manuscript preparation in Month 2).

## Sample size:

Based on a pilot prevalence of 10%, a confidence interval of 95% and a precision of 6%, a final sample size of 106 was calculated. A pilot study was conducted to know the prevalence of knowledge among health care professionals which came out to be 10%. Using this knowledge prevalence and assuming a confidence interval of 95%, absolute precision of 5% and a non-response rate of 10%, the final sample size calculated was 103.

Sample size (n) = (z^2^pq)/l^2^

Z=1.96

p=prevalence

q= 100 - p

l= allowable error

p=10

q=100-10

z=1.96 (95% CI)

l=error (6%)

n= (1.96 * 1.96 * 10 * 90) / 36

n = 96 (approx.) + 10% (non-responders) = 106

prevalence=10% (approx.) (11)

Of 300 invited participants, 107 responded.

## Study procedure:

A pre-validated 15-item KAP questionnaire(7) was distributed electronically among the professionals via email or WhatsApp (Meta Platforms, Inc., Menlo Park, California, United States). Close-ended questions having a simple "yes" or "no" as answer options were used to evaluate the knowledge, attitude and practice of materiovigilance among the healthcare professionals

## Data collection and analysis: 

Responses were recorded electronically after obtaining informed consent from participants. Data were entered into Microsoft Excel version 2023 (Microsoft Corporation, Redmond, Washington, United States) and analysed using SPSS (version 21). The chi-square test was applied for categorical comparisons; p<0.05 was considered statistically significant.

## Results:

Out of 107 respondents, 28 were specialists, 13 were midwifery staff, 14 were medical officers and 52 were nursing staff. The response rate was 36%. Table 1 reveals that 58% of participants had heard of materiovigilance. Awareness of medical devices causing adverse events was highest among medical officers (100%) and specialists (96%), followed by nursing staff (90%) and midwifery staff (77%), with no statistically significant difference (P=0.118). A similar pattern was observed in knowledge about reporting such events, with 100% of medical officers, 90% of nursing staff, 77% of midwifery staff and 75% of specialists aware that medical device- related adverse events (MDAEs) can be reported. Only 43% of specialists and 40% of nurses knew where to report MDAEs. Nearly all respondents (96.2%) believed MDAE reporting is essential for patient safety. Of these, 28 (100%) were specialists, 12 (92%) were midwifery staff, 12 (86%) were medical officers and 48 (92%) were nursing staff (p = 0.324). A majority favoured inclusion of materiovigilance in the medical curriculum (89.2%) (p = 0.136) and supported the expansion of MDMCs as depicted in Table 2. 35% had encountered an MDAE during their practice (P=0.033), which is statistically significant, yet only 14.9% had reported it. Attendance at MvPI workshops or CMEs was low (18%). Statistically significant gaps were observed between knowledge and reporting practices (p=0.027). Detailed data of practice-based questions are shown in Table 3.

## Discussion:

Materiovigilance is no longer a novel concept in India; the Materiovigilance Programme of India (MvPI) was officially launched in July 2015 by the Drugs Controller General of India under the coordination of the Indian Pharmacopoeia Commission [9]. Despite the programme being operational for nearly a decade, findings from this study reveal critical gaps in knowledge, awareness and practice among healthcare professionals in Central India. These results align with the sparse literature available from the region and highlight the need for intensified efforts to integrate materiovigilance into routine clinical practice. Although 60% of respondents in our study had heard of the term "materiovigilance", only 31% had ever seen the official Medical Device Adverse Event (MDAE) reporting form. This mirrors findings from Meher et al. where awareness was similarly superficial despite the long-standing availability of the reporting system [10]. The limited exposure to essential reporting tools suggest a need for widespread sensitization and training initiatives. Encouragingly, most participants acknowledged the importance of MDAE reporting: 93% agreed it is essential and 96% believed it enhances patient safety. These findings are consistent with a study by Meher *et al.* where a positive attitude was also documented among healthcare workers towards medical device safety reporting [11]. Nearly half of them (55%) had the view that one materiovigilance centre is enough for a city. Furthermore, a significant proportion supported integrating materiovigilance into the undergraduate medical curriculum (88%)-a step that could institutionalize reporting habits early in medical training and mirror global best practices. However, translating positive attitudes into consistent practice remains a challenge. Only 35% of participants had encountered a medical device-related adverse event during their professional experience and of these, a mere 13% proceeded to report it. Additionally, 82% of respondents had never attended any continuing medical education (CME) program or workshop on materiovigilance-a figure comparable to the 91% reported by Meher *et al.* [11]. This suggests that current professional development efforts are insufficient and may not adequately target all cadres of healthcare workers. Lack of regular, structured training perpetuates underreporting and limits system responsiveness. Bhosale *et al.* [12] also reported considerable deficiencies in awareness and practical understanding of materiovigilance among healthcare professionals, emphasizing that inadequate training and limited exposure to reporting protocols significantly contribute to underreporting of medical device adverse events. Similarly, Sharon *et al.* [13] observed that although healthcare workers generally demonstrate a positive attitude toward patient safety and adverse event surveillance, actual reporting practices remain poor due to insufficient institutional support, lack of awareness regarding reporting procedures and inadequate educational interventions. In addition, Sojitra *et al.* [14] and Selvam *et al.* (2023) [15] identified several barriers responsible for low reporting rates, including uncertainty in causality assessment, fear of legal implications, time constraints and lack of familiarity with established reporting systems. These findings collectively support the observations of the present study and highlight the urgent need for structured training programs, regular continuing medical education activities and simplified reporting mechanisms to strengthen materiovigilance practices among healthcare professionals.

## Conclusion:

We show that although attitudes are largely favourable, there is a disconnect between awareness and practical engagement with the MvPI framework. Structured CME programmes, easily accessible reporting tools and institutional incentives may help bridge this gap. Moreover, focused capacity-building at newly designated MDMCs, like our institute, is essential for developing a culture of proactive device surveillance. This study, among the first from Central India, provides critical insights into the prevailing KAP landscape and serves as a foundation for regional and national efforts aimed at strengthening materiovigilance infrastructure and compliance.

## Figures and Tables

**Table 1:** Participants' responses based on knowledge-based questions

**Q. no**	**Questions**	**Group I (n=28), % of total**	**Group II (n=13), % of total**	**Group III (n=14), % of total**	**Group IV (n=52), % of total**	**P**
1	Have you heard the term-Materiovigilance?	17(61)	09(69)	11(79)	28(54)	0.384
2	Do you know that medical devices (for example suture material, infusion set, nasogastric tube, intradermal patch, ortho implant) can cause adverse events?	27(96)	10(77)	14(100)	47(90)	0.118
3	Do you know that medical device related adverse events can be reported?	21(75)	04(31)	12(86)	44(85)	0.497
4	Do you know where to report medical device related adverse event?	12(43)	08(62)	09(64)	21(40)	0.274
5	Have you seen the medical device related adverse event (MDAE) reporting form?	07(25)	05(38)	05(36)	16(31)	0.811

**Table 2:** Participants' responses based on attitude-based questions

**Q. no**	**Questions**	**Group I (n=28), % of total**	**Group II (n=13), % of total**	**Group III (n=14), % of total**	**Group IV (n=52), % of total**	**P**
1	Medical device related adverse event reporting is essential?	28(100)	12(92)	12(86)	48(92)	0.324
2	Do you think medical device related adverse event reporting will enhance safety of patients?	28(100)	12(92)	14(100)	49(94)	0.425
3	One materio-vigilance center is enough for a city?	14(50)	05(38)	08(57)	28(54)	0.788
4	Every medical institute should enroll under materiovigilance program?	28(100)	11(85)	14(100)	49(94)	0.136
5	Materiovigilance should be included in the undergraduate curriculum?	27(96)	09(69)	12(86)	46(88)	0.101

**Table 3:** Participants' responses based on practice-based questions

**Q. no**	**Questions**	**Group I (n=28), % of total**	**Group II (n=13), % of total**	**Group III (n=14), % of total**	**Group IV (n=52), % of total**	**P**
1	Have you ever come across an adverse event related to medical device during your practice?	10(36)	08(62)	07(50)	12(23)	0.033
2	If yes, have you reported that?	04(14)	05(38)	01(07)	04(08)	0.027
3	Have you documented any medical device related adverse event?	03(11)	07(54)	01(07)	04(08)	0
4	Have you attended any CME/workshop/seminar on materiovigilance?	07(25)	01(08)	03(21)	07(13)	0.433
5	Have you ever taken any feedback for any untoward event from patients after implanting a medical device?	03(11)	07(54)	04(29)	12(23)	0.027
